# Supplementary material for: The Polycomb system sustains promoters in a deep OFF state by limiting pre-initiation complex formation to counteract transcription
Source: Nat Cell Biol. 2024 Sep 11;26(10):1700–11. doi: 10.1038/s41556-024-01493-w (PMC11469961; doi:10.1038/s41556-024-01493-w)
Supplement: Supplementary file 1 — Reporting Summary [file 41556_2024_1493_MOESM1_ESM.pdf]

Corresponding author(s): Robert J. Klose
Aleksander T. Szczurek

Last updated by author(s): 28/02/2024

Reporting Summary

Nature Portfolio wishes to improve the reproducibility of the work that we publish. This form provides structure for consistency and transparency in reporting. For further information on Nature Portfolio policies, see our Editorial Policies and the Editorial Policy Checklist.

Statistics

For all statistical analyses, confirm that the following items are present in the figure legend, table legend, main text, or Methods section.

n/a	Confirmed	
☐	☒	The exact sample size (*n*) for each experimental group/condition, given as a discrete number and unit of measurement
☐	☒	A statement on whether measurements were taken from distinct samples or whether the same sample was measured repeatedly
☐	☒	The statistical test(s) used AND whether they are one- or two-sided<br>*Only common tests should be described solely by name; describe more complex techniques in the Methods section.*
☒	☐	A description of all covariates tested
☒	☐	A description of any assumptions or corrections, such as tests of normality and adjustment for multiple comparisons
☐	☒	A full description of the statistical parameters including central tendency (e.g. means) or other basic estimates (e.g. regression coefficient) AND variation (e.g. standard deviation) or associated estimates of uncertainty (e.g. confidence intervals)
☐	☒	For null hypothesis testing, the test statistic (e.g. *F*, *t*, *r*) with confidence intervals, effect sizes, degrees of freedom and *P* value noted<br>*Give P values as exact values whenever suitable.*
☒	☐	For Bayesian analysis, information on the choice of priors and Markov chain Monte Carlo settings
☒	☐	For hierarchical and complex designs, identification of the appropriate level for tests and full reporting of outcomes
☐	☒	Estimates of effect sizes (e.g. Cohen's *d*, Pearson's *r*), indicating how they were calculated

Our web collection on statistics for biologists contains articles on many of the points above.

Software and code

Policy information about availability of computer code

Data collection	Image Studio v5.2 (LI-COR), CellSens v.1.17 (Olympus)
Data analysis	R version 4.0.2 (R Core Team, 2020), Bowtie 2 (Langmead and Salzberg, 2012), STAR v2.5.4(Dobin et al., 2013), MACS2 v2.1.1 (Zhang et al., 2008), Sambamba 0.6.7 (Tarasov et al., 2015), SAMtools v1.7 (Li et al., 2009), deepTools v3.1.1 (Ramirez et al., 2016), BEDtools v2.17.0 (Quinlan and Hall, 2010), DESeq2 (Love et al., 2014), ImageJ 1.52p through Fiji (Schindelin et al., 2012), Matlab 2013b (Mathworks), Spot-ON v.1 (Hansen et al., 2018)

For manuscripts utilizing custom algorithms or software that are central to the research but not yet described in published literature, software must be made available to editors and reviewers. We strongly encourage code deposition in a community repository (e.g. GitHub). See the Nature Portfolio guidelines for submitting code & software for further information.

Data

Policy information about availability of data

All manuscripts must include a data availability statement. This statement should provide the following information, where applicable:

- Accession codes, unique identifiers, or web links for publicly available datasets
- A description of any restrictions on data availability
- For clinical datasets or third party data, please ensure that the statement adheres to our policy

The high-throughput sequencing datasets generated for this study are publically available in the GEO database under GSE216636 accession number (https://www.ncbi.nlm.nih.gov/geo/query/acc.cgi?acc=GSE216636). Localised single molecule positions obtained in particle tracking experiments can be provided upon request. Numerical files for transcription activity trajectories are available upon request. For cnRNA-seq processing we used mm10 (GenBank: BK000964.3, https://www.ncbi.nlm.nih.gov/nuccore/bk000964) and dm6 (GenBank: M21017.1, https://www.ncbi.nlm.nih.gov/nuccore/M21017.1) rDNA genomic datasets.

Research involving human participants, their data, or biological material

Policy information about studies with human participants or human data. See also policy information about sex, gender (identity/presentation), and sexual orientation and race, ethnicity and racism.

Reporting on sex and gender	n.a.
Reporting on race, ethnicity, or other socially relevant groupings	n.a.
Population characteristics	n.a.
Recruitment	n.a.
Ethics oversight	n.a.

Note that full information on the approval of the study protocol must also be provided in the manuscript.

Field-specific reporting

Please select the one below that is the best fit for your research. If you are not sure, read the appropriate sections before making your selection.

☒ Life sciences ☐ Behavioural & social sciences ☐ Ecological, evolutionary & environmental sciences

For a reference copy of the document with all sections, see nature.com/documents/nr-reporting-summary-flat.pdf

Life sciences study design

All studies must disclose on these points even when the disclosure is negative.

Sample size	Sample sizes were determined based on previous studies using similar techniques to enable reasonable statistical analysis and reproducibility (https://doi.org/10.1038/s41467-021-21130-6). Cell counts for RNA-FISH were previously determined by us (https://doi.org/10.1038/s41594-021-00661-y). Numbers of cells measured in live-cell transcription measurements were large enough to ensure consistency and reproducibility between biological replicates. Number of biological replicates for the ChIP-seq experiments was previously determined by us (https://doi.org/10.1038/s41594-022-00840-5).
Data exclusions	No data were excluded
Replication	Reported experimental findings were reproducible in multiple biological replicates. The numbers of biological replicates for each experiment are stated. Vast majority of the biological replicates have been performed within a few days apart.
Randomization	To plot heatmaps of transcriptional activity and to allow direct comparison between conditions the datasets required to be of the same size. In order to acheive that the data-sets was randomly subsampled to present the same number of cells per condition and hence allow direct qualitative comparison of different conditions as heatmaps (Figure. 3e and Extended Data Figure 8b). cChIP-data were randomly subsampled as mentioned y us previously (https://doi.org:10.1016/j.molcel.2019.03.024). The number of ON-periods per Permissive period in extended Data Figure 4d was assumed to follow Poisson distribution and was randomly drawn from this distribution for the sake of transcription simulations.
Blinding	All the imaging experiments have been performed and analysed by a single person and were therefore not blinded.

Behavioural & social sciences study design

All studies must disclose on these points even when the disclosure is negative.

Study description	*Briefly describe the study type including whether data are quantitative, qualitative, or mixed-methods (e.g. qualitative cross-sectional, quantitative experimental, mixed-methods case study).*
Research sample	*State the research sample (e.g. Harvard university undergraduates, villagers in rural India) and provide relevant demographic information (e.g. age, sex) and indicate whether the sample is representative. Provide a rationale for the study sample chosen. For studies involving existing datasets, please describe the dataset and source.*
Sampling strategy	*Describe the sampling procedure (e.g. random, snowball, stratified, convenience). Describe the statistical methods that were used to predetermine sample size OR if no sample-size calculation was performed, describe how sample sizes were chosen and provide a rationale for why these sample sizes are sufficient. For qualitative data, please indicate whether data saturation was considered, and what criteria were used to decide that no further sampling was needed.*
Data collection	*Provide details about the data collection procedure, including the instruments or devices used to record the data (e.g. pen and paper, computer, eye tracker, video or audio equipment) whether anyone was present besides the participant(s) and the researcher, and whether the researcher was blind to experimental condition and/or the study hypothesis during data collection.*
Timing	*Indicate the start and stop dates of data collection. If there is a gap between collection periods, state the dates for each sample cohort.*
Data exclusions	*If no data were excluded from the analyses, state so OR if data were excluded, provide the exact number of exclusions and the rationale behind them, indicating whether exclusion criteria were pre-established.*
Non-participation	*State how many participants dropped out/declined participation and the reason(s) given OR provide response rate OR state that no participants dropped out/declined participation.*
Randomization	*If participants were not allocated into experimental groups, state so OR describe how participants were allocated to groups, and if allocation was not random, describe how covariates were controlled.*

Ecological, evolutionary & environmental sciences study design

All studies must disclose on these points even when the disclosure is negative.

Study description	*Briefly describe the study. For quantitative data include treatment factors and interactions, design structure (e.g. factorial, nested, hierarchical), nature and number of experimental units and replicates.*
Research sample	*Describe the research sample (e.g. a group of tagged Passer domesticus, all Stenocereus thurberi within Organ Pipe Cactus National Monument), and provide a rationale for the sample choice. When relevant, describe the organism taxa, source, sex, age range and any manipulations. State what population the sample is meant to represent when applicable. For studies involving existing datasets, describe the data and its source.*
Sampling strategy	*Note the sampling procedure. Describe the statistical methods that were used to predetermine sample size OR if no sample-size calculation was performed, describe how sample sizes were chosen and provide a rationale for why these sample sizes are sufficient.*
Data collection	*Describe the data collection procedure, including who recorded the data and how.*
Timing and spatial scale	*Indicate the start and stop dates of data collection, noting the frequency and periodicity of sampling and providing a rationale for these choices. If there is a gap between collection periods, state the dates for each sample cohort. Specify the spatial scale from which the data are taken*
Data exclusions	*If no data were excluded from the analyses, state so OR if data were excluded, describe the exclusions and the rationale behind them, indicating whether exclusion criteria were pre-established.*
Reproducibility	*Describe the measures taken to verify the reproducibility of experimental findings. For each experiment, note whether any attempts to repeat the experiment failed OR state that all attempts to repeat the experiment were successful.*
Randomization	*Describe how samples/organisms/participants were allocated into groups. If allocation was not random, describe how covariates were controlled. If this is not relevant to your study, explain why.*
Blinding	*Describe the extent of blinding used during data acquisition and analysis. If blinding was not possible, describe why OR explain why blinding was not relevant to your study.*

Did the study involve field work? ☐ Yes ☒ No

Reporting for specific materials, systems and methods

We require information from authors about some types of materials, experimental systems and methods used in many studies. Here, indicate whether each material, system or method listed is relevant to your study. If you are not sure if a list item applies to your research, read the appropriate section before selecting a response.

Materials & experimental systems

n/a	Involved in the study
☐	☒ Antibodies
☐	☒ Eukaryotic cell lines
☒	☐ Palaeontology and archaeology
☒	☐ Animals and other organisms
☒	☐ Clinical data
☒	☐ Dual use research of concern
☒	☐ Plants

Methods

n/a	Involved in the study
☐	☒ ChIP-seq
☒	☐ Flow cytometry
☒	☐ MRI-based neuroimaging

Antibodies

Antibodies used

anti-RING1B Cell Signaling Cat# 5694, Western
anti-TBP Abcam Cat# ab818, Western
anti-T7 Cell Signalling Cat# 13246, D9E1X, Western, ChIP
anti-SUZ12 Cell Signalling Cat# 3737, Western
anti-Cdk7 Thermofisher Cat# 31TF2-1F8, Western
anti-TFIIB Abcam Cat# ab109106, Western
anti-TAF1 Thermofisher Cat# PA5-104490, Western
anti-TAF11 Proteintech Cat# 16114-1-AP, Western
anti-NC2b Santa Cruz Biotechnology Cat# SC-515024, Western
anti-Rpb1 Cell Signalling Cat# 14958, Western
anti-Cdk9 Abcam Cat# ab239364, Western
anti- NELF-B Cell Signalling Cat#14894S, Western
anti-Brg1, Abcam, Cat# ab110641, Western
anti-PCGF2, Santa Cruz Biotechnology Cat# sc-10744, Western
anti-PCGF2, produced in house, Western
anti-PHC1, Cell Signalling, Cat# 13768, Western
IRDye 800CW Goat anti-Mouse IgG, LI-COR, Cat# 926-32210, Western
IRDye 800CW Goat anti-Rabbit IgG, LI-COR, Cat# 926-32211, Western
IRDye 680RD Goat anti-Mouse IgG, LI-COR, Cat# 926-68070, Western
IRDye 680RD Goat anti-Rabbit IgG, LI-COR, Cat# 926-68071, Western

Validation

anti-RING1B; verified by us previously (Dobrinic et al., 2021) and in this work in Ring1B degron line
anti-TBP; verified by us by endogenous tagging and molecular weight shift in Western, manufacturer validated in various cell types by cellular fractionation, 276 citations: https://www.citeab.com/antibodies/753557-ab818-anti-tata-binding-protein-tbp-antibody-1tbp18?des=1ee5e4f398055d5b
anti-T7; verified by the manufacturer and by us through Western analysis of nuclear extracts with and withoutT7-tagged protein (Ext. Data Fig. 5a), 19 citations https://www.citeab.com/antibodies/2043128-13246-t7-tag-d9e1x-xp-rabbit-mab?des=770a8fbd62dd70ee
anti-SUZ12; manufacturer-validated against various cell lines by Western blot, verified by us previously in Suz12-degron line (Dobrinic et al., 2021)
anti-Cdk7; verified by us by endogenous tagging and molecular weight shift in Western, verified by manufactured by knockdown
anti-TFIIB; verified by us by endogenous degron tagging, band molecular weight shift and disappearing in Western analysis
anti-TAF1; validated here in TAF1 degron line tagged with T7-tag and by molecular weight shift in Western (Ext. Data Fig. 5a)
anti-TAF11; verified by us by endogenous degron tagging, band molecular weight shift and disappearing in Western analysis
anti-NC2b; verified by us by endogenous tagging and molecular weight shift in Western and degron tagging.
anti-Rpb1; verified by us by endogenous tagging and molecular weight shift in Western and by ChIP-seq previously (Dorbinic et al., 2021)
anti-Cdk9; verified by us by endogenous tagging and molecular weight shift and presence of second band corresponding to phosphorylation
anti-NELF-B; verified by us by endogenous degron tagging, band molecular weight shift and disappearing in Western analysis
anti-Brg1; verified by the manufacturer in knockdown and knockout line and by us by endogenous tagging and Western analysis, 213 citations, https://www.citeab.com/antibodies/715090-ab110641-anti-brg1-antibody-epncir111a?des=cf05e3cd167c6000
anti-PCGF2 (sc); verified by the manufacturer and by WB in a degron line
anti-PCGF2 (custom); verified by WB and ChIPseq in a conditional knock-out line and in two degron lines (this work, Fig. 5)
anti-PHC1; verified by the manufacturer and by us in Pcgf2-degron lines where its levels decrease upon Pcgf2 depletion (Fig. 5b, d)

Eukaryotic cell lines

Policy information about cell lines and Sex and Gender in Research

Cell line source(s)	All mouse embryonic stem cell lines used in this study were generated in the Klose lab and were in Ring1B-AID/Ring1A-/- background extensively characterised by us in Dobrinic et al., 2021:<br>MCP-GFP/Rosa26<br>MCP-GFP/Rosa26 MS2x128-Zic2<br>MCP-GFP/Rosa26 MS2x128-E2f6<br>MCP-GFP/Rosa26 MS2x128-Hspg2<br>MCP-GFP/Rosa26 MS2x128-Meis1<br>T7-dTAG-TAF1<br>T7-HaloTag-dTAG-TAF1<br>HaloTag-NC2b<br>HaloTag-TBP<br>HaloTag-Rpb1<br>HaloTag-TAF11<br>Cdk7-HaloTag<br>T7-HaloTag-TFIIB<br>HaloTag-Cdk9<br>NELF-B-HaloTag<br>T7-HaloTag-MED14<br>T7-dTAG-TAF1/PCGF2-bromoTAG<br>HaloTag-TAF11/PCGF2-dTAG<br>Human HEK293T or drosophila SG4 cells (sourced from ATCC) were used as material for calibration but not as an experimental system.
Authentication	Each cell line was validated at genetic level by PCR flanking the desired insertion site, and on protein level by Western blot analysis, and by microscopy to test nuclear localisation HaloTag protein tagging. MS2 cell lines were validated for proper array insertion by microscopy and RNA-FISH against intronic sequences meant to colocalize with MCP-GFP signal accumulation.
Mycoplasma contamination	All cell lines were regularly tested for mycoplasma contamination and confirmed to be negative.
Commonly misidentified lines<br>(See ICLAC register)	No commonly misidentified lines were used in this work

Plants

Seed stocks	Not applicable.
Novel plant genotypes	Not applicable.
Authentication	Not applicable.

ChIP-seq

Data deposition

☒ Confirm that both raw and final processed data have been deposited in a public database such as GEO.

☒ Confirm that you have deposited or provided access to graph files (e.g. BED files) for the called peaks.

Data access links<br>*May remain private before publication.*	https://www.ncbi.nlm.nih.gov/geo/query/acc.cgi?acc=GSE216636
Files in database submission	GSM6685040 RING1BmAID-TAF1dTAG-T7-UNT-rep1<br>GSM6685041 RING1BmAID-TAF1dTAG-T7-UNT-rep2<br>GSM6685042 RING1BmAID-TAF1dTAG-T7-UNT-rep3<br>GSM6685046 RING1BmAID-TAF1dTAG-T7-AUX-rep1<br>GSM6685047 RING1BmAID-TAF1dTAG-T7-AUX-rep2<br>GSM6685048 RING1BmAID-TAF1dTAG-T7-AUX-rep3<br>GSM6685049 RING1BmAID-TAF1dTAG-T7-Input-UNT-rep1<br>GSM6685050 RING1BmAID-TAF1dTAG-T7-Input-UNT-rep2<br>GSM6685051 RING1BmAID-TAF1dTAG-T7-Input-UNT-rep3<br>GSM6685055 RING1BmAID-TAF1dTAG-T7-Input-AUX-rep1<br>GSM6685056 RING1BmAID-TAF1dTAG-T7-Input-AUX-rep2<br>GSM6685057 RING1BmAID-TAF1dTAG-T7-Input-AUX-rep3
Genome browser session<br>(e.g. UCSC)	No longer applicable

Methodology

Replicates	All ChIP-seq experiments were performed in biological triplicates
Sequencing depth	All libraries were sequenced as 40bp paired-end reads.
Antibodies	anti-T7 Cell Signalling D9E1X, Cat# 13246
Peak calling parameters	All peaks used in this study were published previously (Dobrinic et al, 2021)
Data quality	Quality of ChIP-seq data was assessed by visual inspection of individual replicate bigWig files and comparison with other published data sets, as well as by metaplot, heatmap and correlation analysis using deepTools.
Software	Paired-end reads were aligned to the concatenated mouse (mm10) and spike-in (dm6 for native, hg19 for cross-linked cChIP-seq) genome sequences using Bowtie 2 ("–no-mixed" and "–no-discordant" options). Only uniquely mapped reads were kept for downstream analysis, after removal of PCR duplicates with Sambamba. Genome coverage tracks were generated using the pileup function from MACS2. Metaplot and heatmap analysis of ChIP-seq read density at regions of interest was performed with computeMatrix and plotProfile/plotHeatmap from deepTools.
