# Supplementary material for: The Polycomb system sustains promoters in a deep OFF state by limiting pre-initiation complex formation to counteract transcription
Source: Nat Cell Biol. 2024 Sep 11;26(10):1700–11. doi: 10.1038/s41556-024-01493-w (PMC11469961; doi:10.1038/s41556-024-01493-w)

Source Data File

The Polycomb system sustains promoters in a deep OFF-state by limiting pre-initiation complex formation to counteract transcription

A. Szczurek, E. Dimitrova, J. Kelley, N. Blackledge & R. Klose

Raw blot images

*Areas used for publication indicated with red frames

Figure 2c

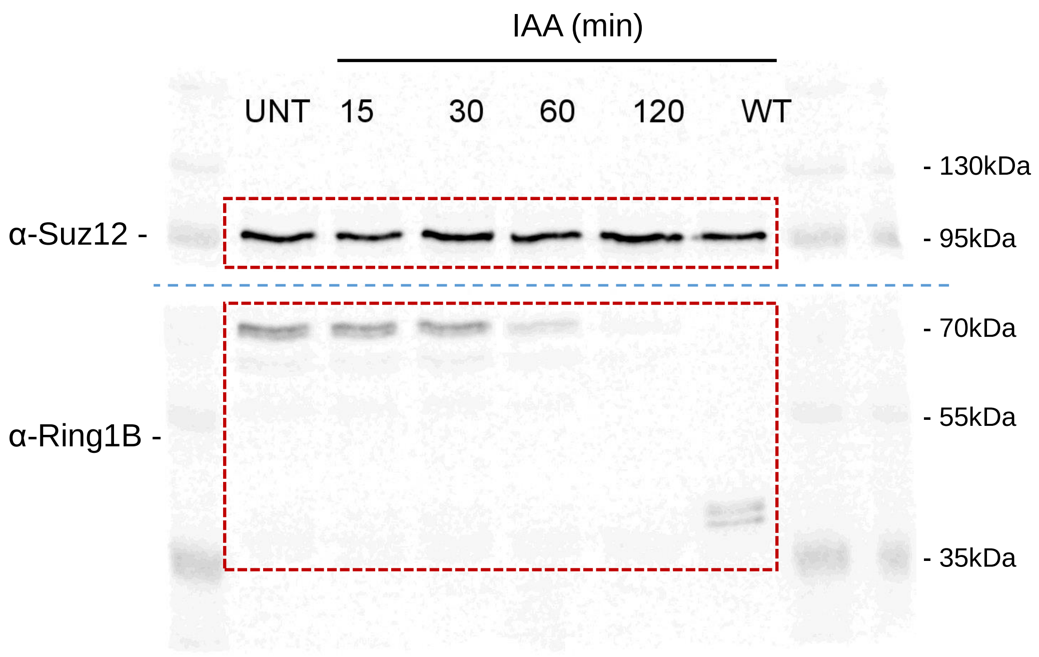

WT – wild type

Figure 5b,d

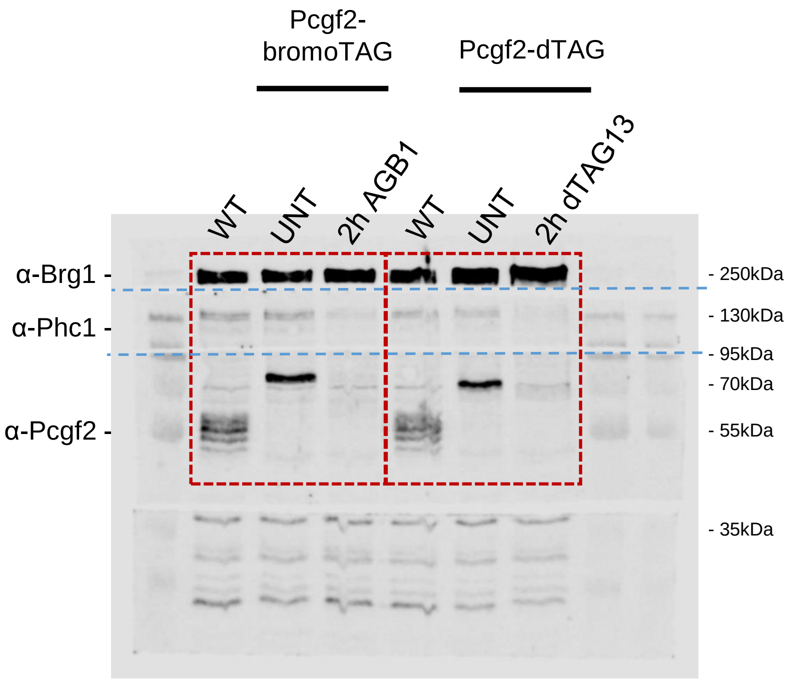

Pcgf2-bromoTAG
Pcgf2-dTAG
WT
UNT
2h AGB1
WT
UNT
2h dTAG13
α-Brg1 -
α-Phc1 -
α-Pcgf2 -
- 250kDa
- 130kDa
- 95kDa
- 70kDa
- 55kDa
- 35kDa

Figure 6d

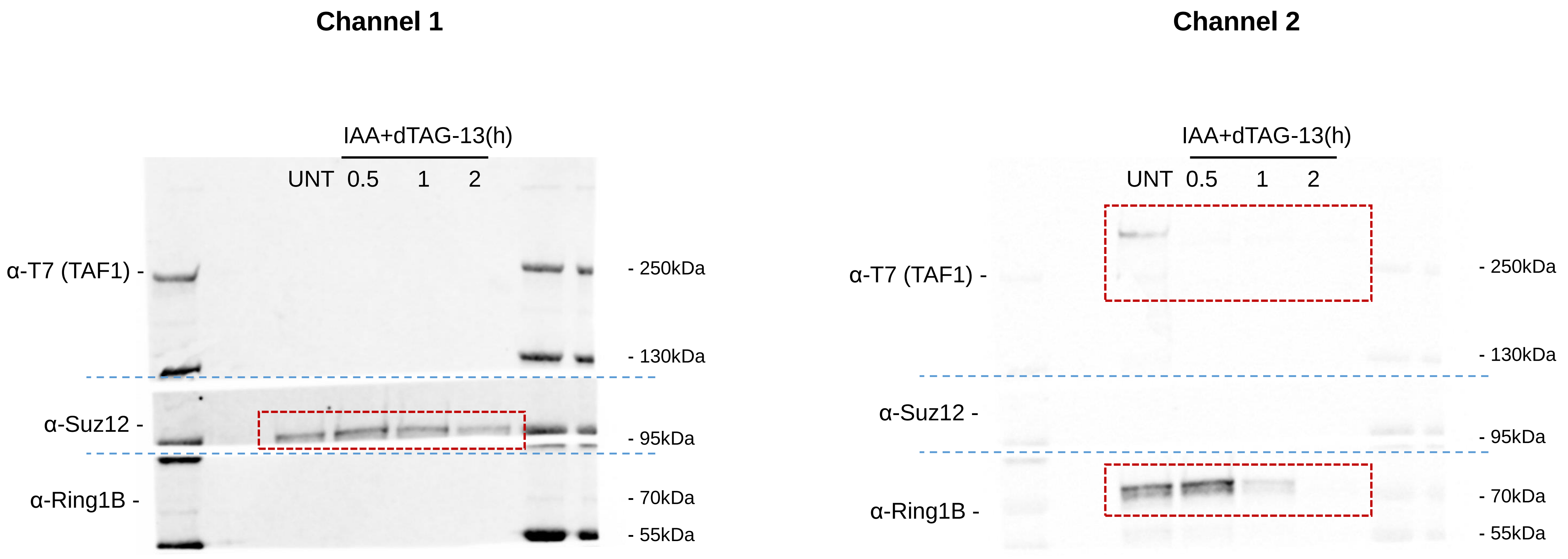

Channel 1
IAA+dTAG-13(h)
UNT 0.5 1 2
α-T7 (TAF1) -
α-Suz12 -
α-Ring1B -
- 250kDa
- 130kDa
- 95kDa
- 70kDa
- 55kDa
Channel 2
IAA+dTAG-13(h)
UNT 0.5 1 2
α-T7 (TAF1) -
α-Suz12 -
α-Ring1B -
- 250kDa
- 130kDa
- 95kDa
- 70kDa
- 55kDa

Extended Data Figure 1a

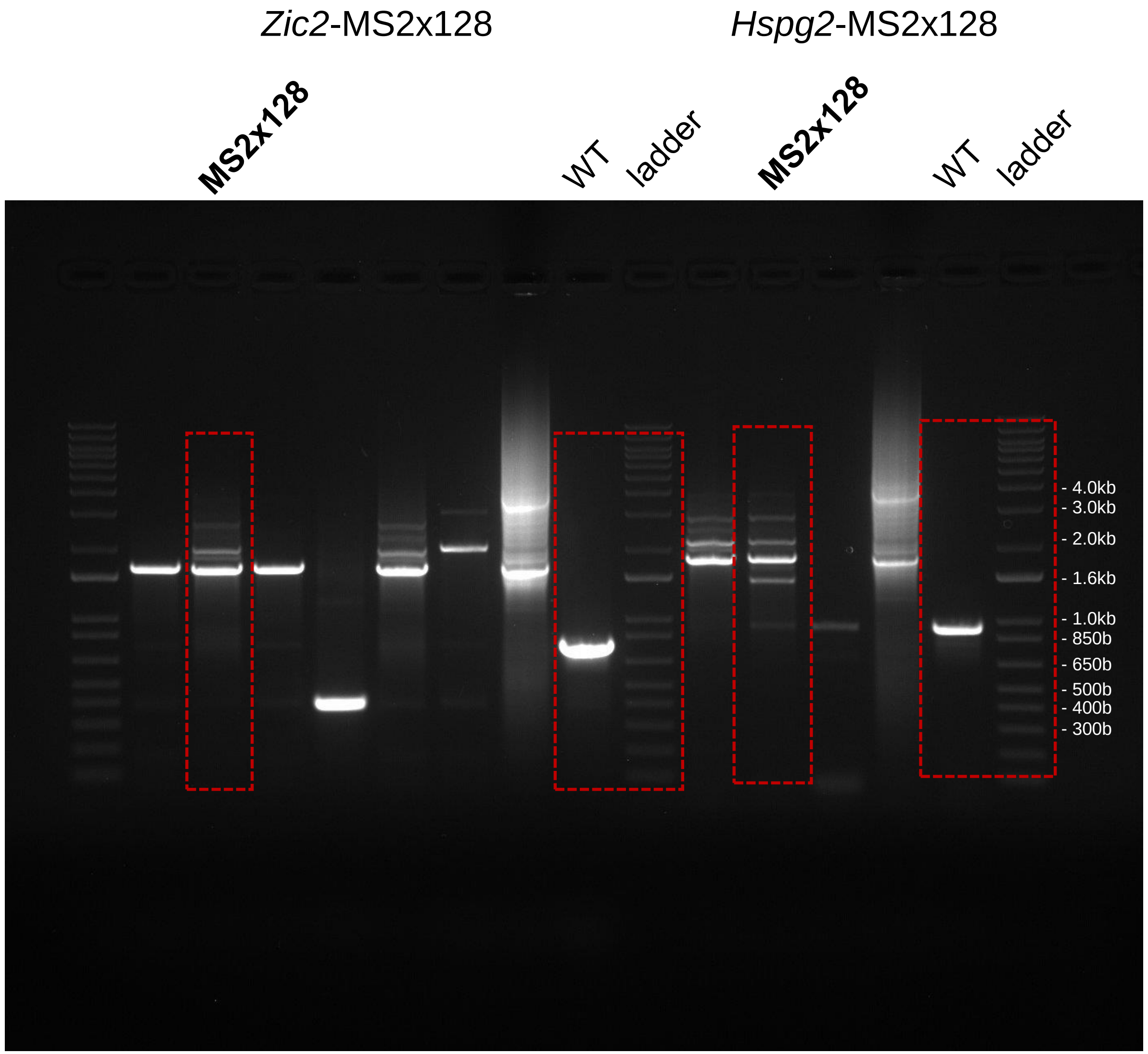

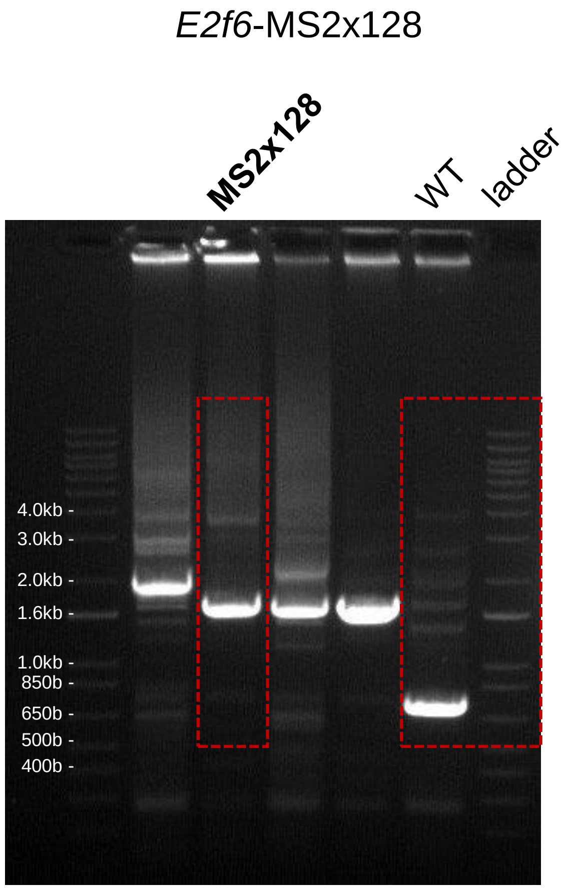

Extended Data Figure 5a – part 1/2

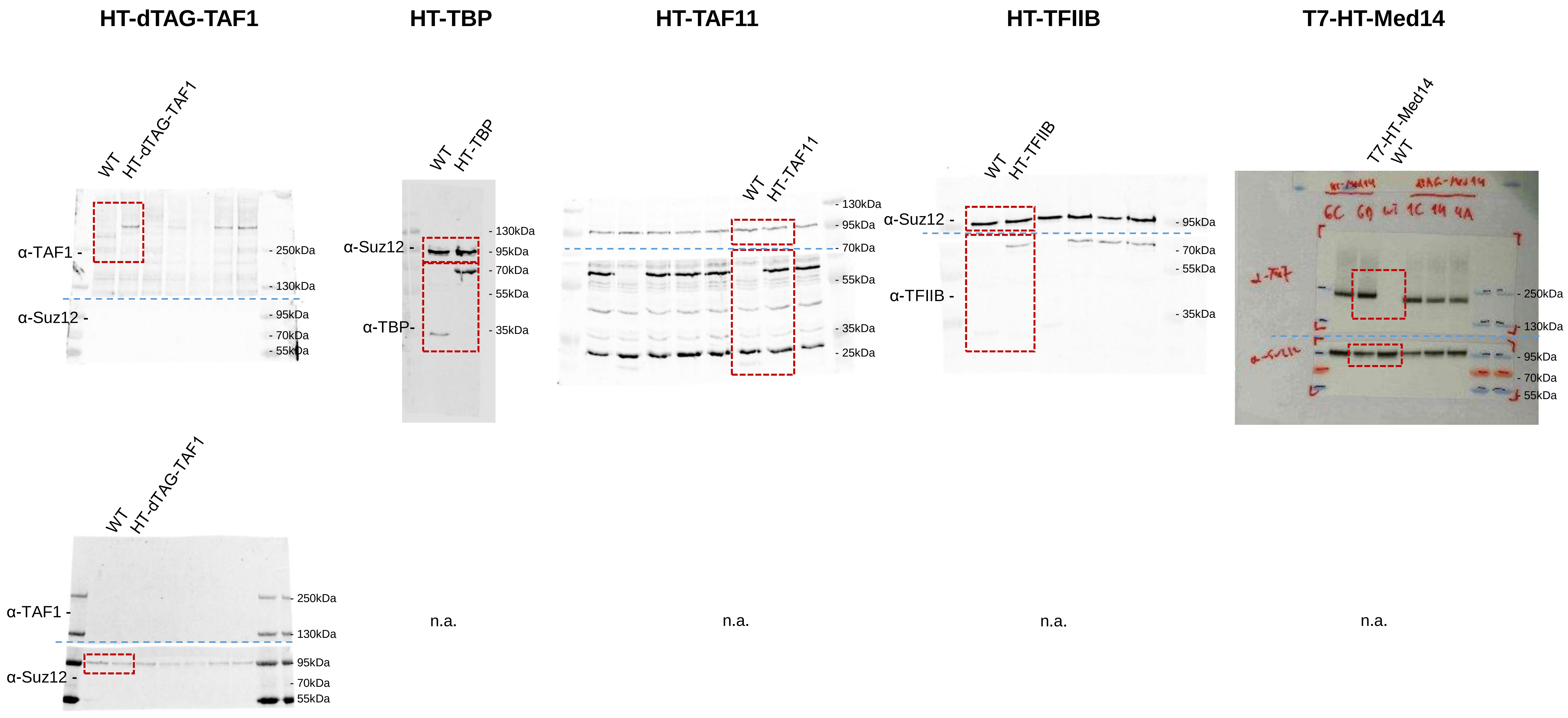

Extended Data Figure 5a – part 2/2

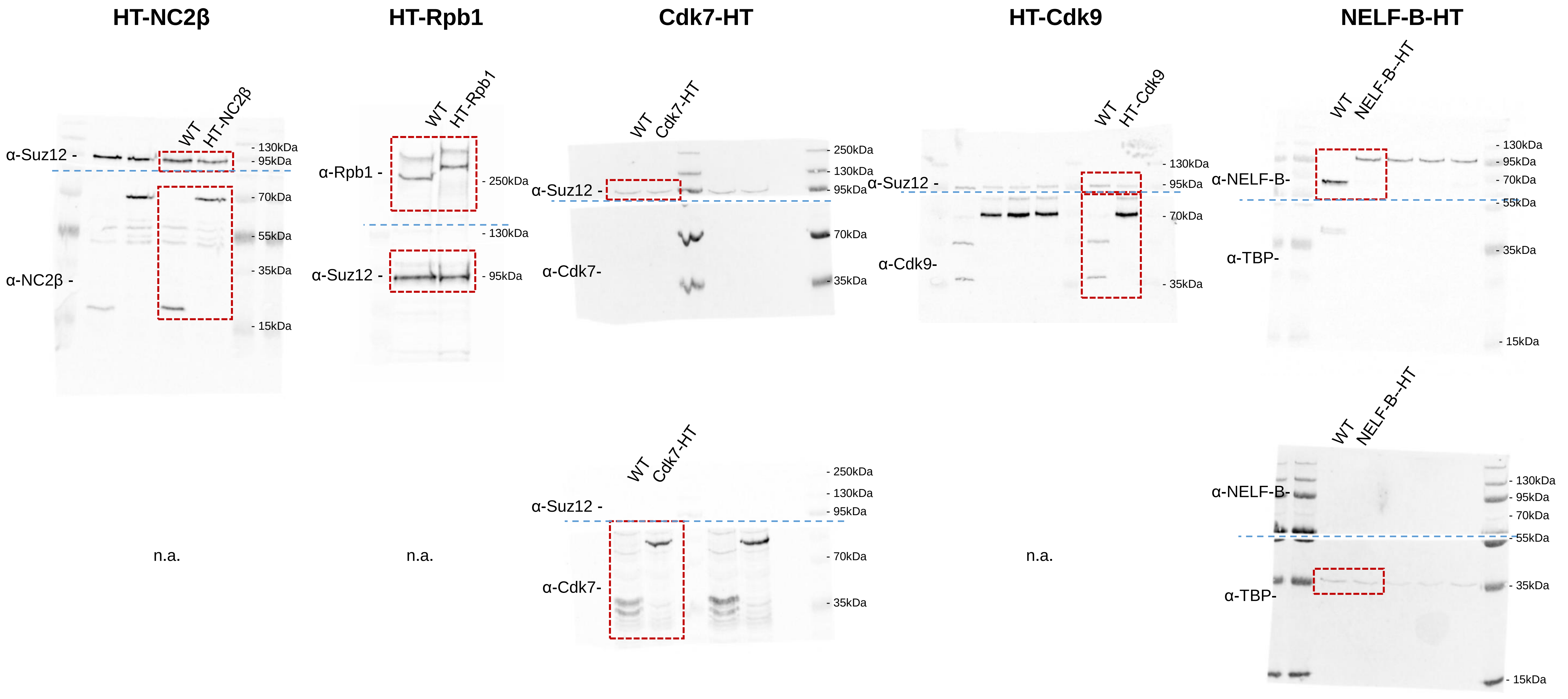

Supplement: Supplementary file 18 — File contains all unprocessed images of blots used in the Article. [file 41556_2024_1493_MOESM18_ESM.pdf]
